# Assessment of databases to determine the validity of β- and γ-carbonic anhydrase sequences from vertebrates

**DOI:** 10.1186/s12864-020-6762-2

**Published:** 2020-05-11

**Authors:** Reza Zolfaghari Emameh, Marianne Kuuslahti, Hassan Nosrati, Hannes Lohi, Seppo Parkkila

**Affiliations:** 1 Department of Energy and Environmental Biotechnology, National Institute of Genetic Engineering and Biotechnology (NIGEB), Tehran, 14965/161 Iran; 2 Faculty of Medicine and Health Technology, Tampere University, FI-33520 Tampere, Finland; 3 Department of Materials Engineering, Tarbiat Modares University, Tehran, Iran; 4 Department of Veterinary Biosciences, University of Helsinki, 00014 Helsinki, Finland; 5 Department of Medical and Clinical Genetics, University of Helsinki, 00014 Helsinki, Finland; 6 Folkhälsan Research Center, 00290 Helsinki, Finland; 7 Fimlab Laboratories Ltd. and Tampere University Hospital, FI-33520 Tampere, Finland

**Keywords:** Carbonic anhydrase, Contamination, Curation, Database, DNA, Sequencing

## Abstract

**Background:**

The inaccuracy of DNA sequence data is becoming a serious problem, as the amount of molecular data is multiplying rapidly and expectations are high for big data to revolutionize life sciences and health care. In this study, we investigated the accuracy of DNA sequence data from commonly used databases using carbonic anhydrase (*CA*) gene sequences as generic targets. CAs are ancient metalloenzymes that are present in all unicellular and multicellular living organisms. Among the eight distinct families of CAs, including α, β, γ, δ, ζ, η, θ, and ι, only α-CAs have been reported in vertebrates.

**Results:**

By an in silico analysis performed on the NCBI and Ensembl databases, we identified several β- and γ-CA sequences in vertebrates, including *Homo sapiens, Mus musculus, Felis catus, Lipotes vexillifer, Pantholops hodgsonii, Hippocampus comes, Hucho hucho, Oncorhynchus tshawytscha, Xenopus tropicalis,* and *Rhinolophus sinicus.* Polymerase chain reaction (PCR) analysis of genomic DNA persistently failed to amplify positive *β-* or *γ-CA* gene sequences when *Mus musculus* and *Felis catus* DNA samples were used as templates. Further BLAST homology searches of the database-derived “vertebrate” β- and γ-CA sequences revealed that the identified sequences were presumably derived from gut microbiota, environmental microbiomes, or grassland ecosystems.

**Conclusions:**

Our results highlight the need for more accurate and fast curation systems for DNA databases. The mined data must be carefully reconciled with our best knowledge of sequences to improve the accuracy of DNA data for publication.

## Background

Carbonic anhydrases (CAs) are metalloenzymes that are classified into eight evolutionarily distinct families, including α, β, γ, δ, ζ, η, θ, and ι [1–4]. These enzymes catalyze the hydration of carbon dioxide to bicarbonate and protons and are involved in various biochemical pathways, such as gluconeogenesis, ureagenesis and photosynthesis, and other physiological functions, such as pH homeostasis, electrolyte transfer and calcification [5].

There are 12 α-CA isozymes, including CA I-IV, CA VA and VB, CA VI, CA VII, CA IX, and CA XII-XIV, that are expressed in humans [6]. Interestingly, CA XV is the only active CA isozyme known to date that is expressed in several vertebrate species but is lost in human and chimpanzee genomes [7]. In addition to the 13 mammalian α-CA isozymes, there are three acatalytic CA-related proteins (CARPs), including CARP VIII, CARP X, and CARP XI, with crucial physiological roles [8–11]. α-CAs have been reported from many organisms, including both prokaryotes and eukaryotes [12].

Although β-CAs are present in archaea, bacteria, plants, fungi, protozoans, and insects, there are no reports of β-CAs in any vertebrate species [13, 14]. Similarly, γ-CAs are present in many prokaryotes and eukaryotes, such as plants and fungi, whereas they do not exist in any vertebrates according to the current knowledge [15, 16]. Incomplete *β-CA* gene sequences have been identified in the genome of the cephalochordate *Branchiostoma floridae* (the Florida lancelet), but whether they represent a pseudogene or an incompletely sequenced active gene has not been determined [17]. Some annotated *β-* and *γ-CA* sequences present in databases have been linked to vertebrate genomes, but in fact, they might have originated from either gut microbiota or other normal flora or even from environmental bacterial contamination. Kraken and Taxoblast are two recently designed ultrafast programs to identify contaminant DNA sequences from metagenomic and genome sequencing databases [18, 19]. The main limitation of both methods is the lack of accessibility to a computer or server with enough RAM for quick operation while performing genome blast homology searches.

In this study, we first searched for β- and γ-CAs in vertebrates using in silico tools. The results obtained from the NCBI and Ensemble databases led us to perform polymerase chain reaction (PCR) amplifications using mouse and cat genomic DNA as templates. The results indicated that the “vertebrate” β- and γ-CA sequences detected from databases were presumably derived from gut microbiota, environmental microbiomes, or grassland ecosystems. This finding emphasizes the importance of fast and accurate biocuration of database sequences.

## Results

### Identification of β- and γ-CAs

**Fig. 1:**
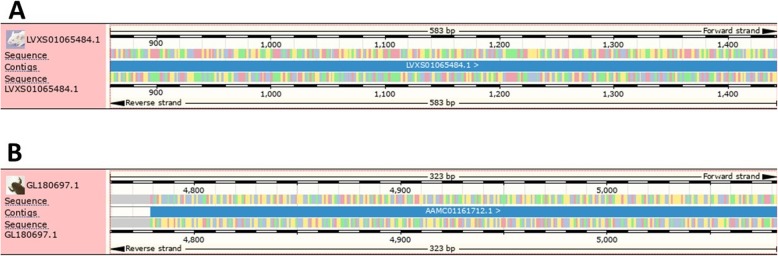
Predicted genomic location of (**a**) a *β-CA* gene in *Mus musculus*, strain NOD/ShiLtJ (scaffold LVXS01065484.1: 870–1430) and (**b**) a *γ-CA* gene in *Xenopus tropicalis* (scaffold GL180697.1: 4765-5075)

**Fig. 2:**
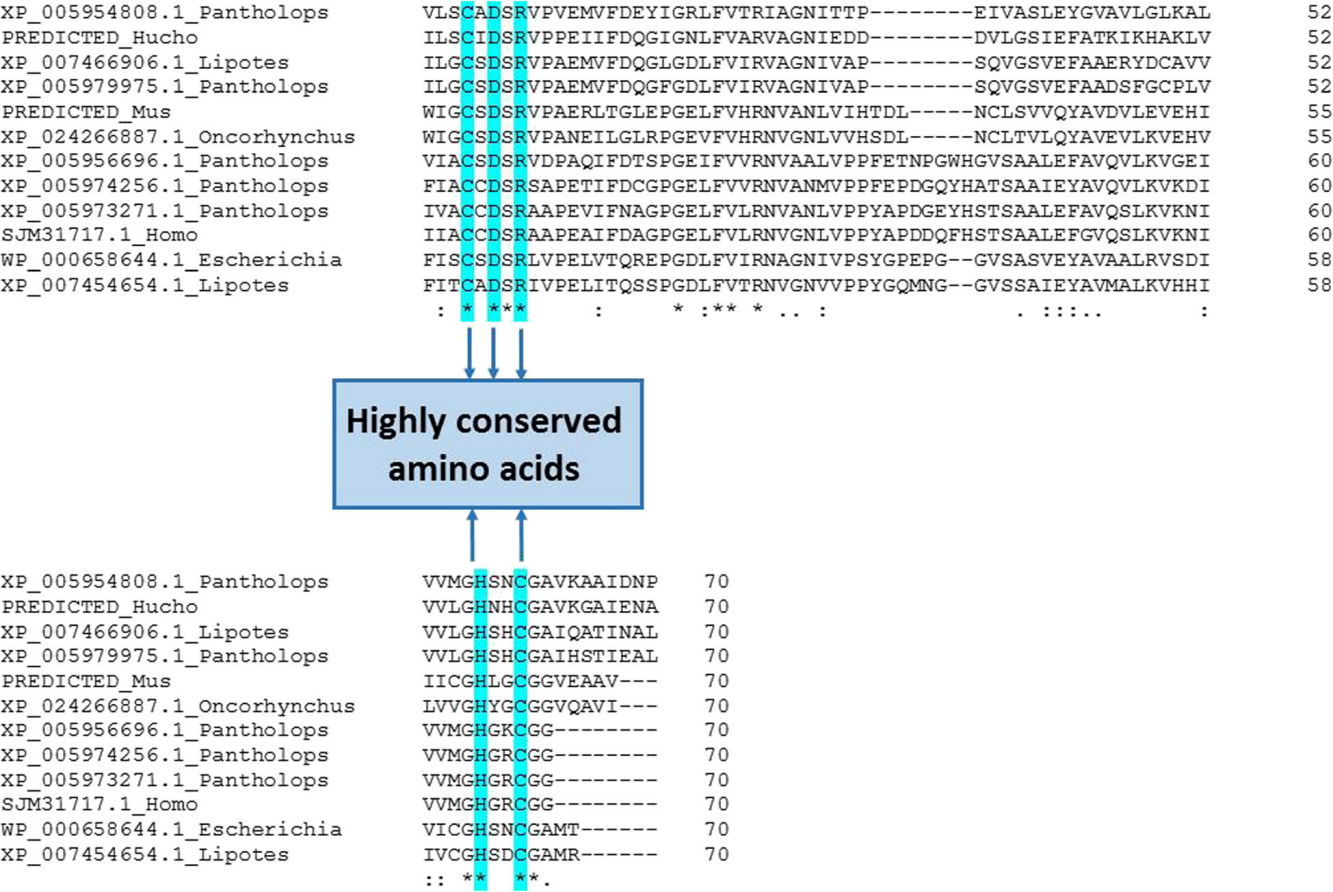
Multiple sequence alignment (MSA) of β-CA protein sequences from vertebrates. The highly conserved amino acids are shown by highlighted vertical bands

**Fig. 3:**
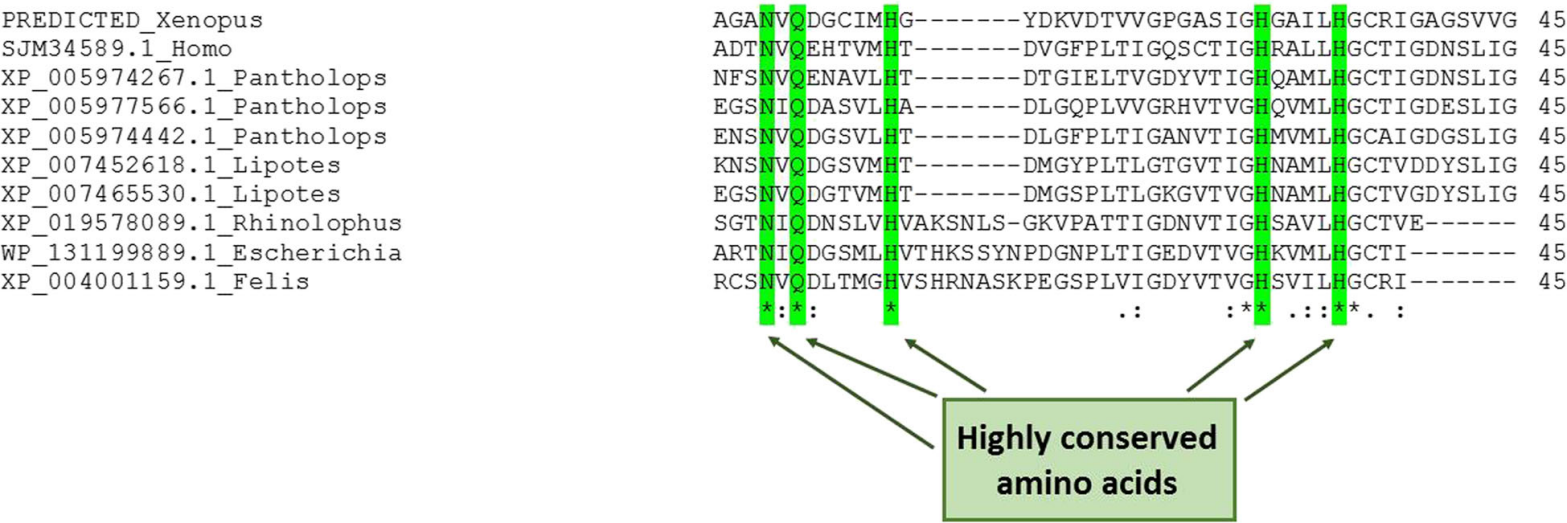
Multiple sequence alignment (MSA) of γ-CA protein sequences from vertebrates. The highly conserved amino acids are shown by highlighted vertical bands

The BLASTP program from the NCBI database identified β-CA protein sequences from some vertebrates, including *Lipotes vexillifer* (XP_007454654.1), *Pantholops hodgsonii* (XP_005974256.1), *Homo sapiens* (SJM31717.1), and *Oncorhynchus tshawytscha* (XP_024266887.1). In addition, the TBLASTN program of Ensembl genome browser 95 identified the genomic location for a *β-CA* gene in *M. musculus*, strain NOD/ShiLtJ (genomic location: LVXS01065484.1: 870–1430), *Hippocampus comes* (genomic location: LVHJ01039623:18–230*), and *Hucho hucho* (genomic location: QNTS01034426:189–644*). The aforementioned methods identified γ-CA protein sequences from some vertebrates, including *L. vexillifer* (XP_007452618.1), *P. hodgsonii* (XP_005961532.1), *H. sapiens* (SJM34589.1), *F. catus* (XP_004001159.1), and *Rhinolophus sinicus* (XP_019578089.1). Additionally, the genomic location was identified for a *γ-CA* gene in *Xenopus tropicalis* (genomic location: GL180697.1: 4765-5075) and *H. comes* (genomic location: LVHJ01047219:4–240*) (Fig. 1 and Table 1). The multiple sequence alignment (MSA) analysis showed that the predicted polypeptide sequences would contain highly conserved amino acids, which are considered important for the classical β-CA (Fig. 2) and γ-CA (Fig. 3) enzymes.

**Table 1:** Identified β-and γ-CAs from vertebrates

Type of CA	NCBI IDs	Vertebrate species	Status in database		73–100% identical species	Exon count
			2017–2018	2019–2020		
**β-CA**	XP_007454654.1	*Lipotes vexillifer* (extinct Yangtze River dolphin)	A	A	*Pseudomonas* sp.	1
	XP_007466906.1				*Acinetobacter* sp.	
	XP_005974256.1	*Pantholops hodgsonii* (Tibetan antelope)	A	D	*Agrobacterium* sp. *Rhizobium* sp.	1
	XP_005956696.1				*Sphingobium* sp.	
	XP_005973271.1				*Mesorhizobium* sp.	
	XP_005979975.1				*Acinetobacter* sp.	
	XP_005954808.1				*Sphingobium* sp.	
	LVXS01065484.1: 870–1430^a^	*Mus musculus*, strain NOD/ShiLtJ (house mouse)	A	A	*Serratia* sp.	ND
	SJM31717.1	*Homo sapiens* (Human)	A	D	*Mesorhizobium delmotii*	1
	LVHJ01039623:18–230^a^	*Hippocampus comes*^b^ (Tiger tail seahorse)	U	A	*Muricauda* sp. (87.3%)	ND
	QNTS01034426:189–644^a^	*Hucho hucho* (Huchen or Danube salmon)	U	A	*Flavobacterium* sp. (73.7%)	ND
	XP_024266887.1	*Oncorhynchus tshawytscha* (Chinook salmon)	U	A	*Hydrogenophaga* sp.	1
**γ-CA**	XP_007452618.1	*Lipotes vexillifer* (extinct Yangtze River dolphin)	A	A	*Pseudomonas* sp.	1
	XP_007465530.1					
	XP_005974442.1	*Pantholops hodgsonii* (Tibetan antelope)	A	D	*Caulobacter* sp.	1
	XP_005977566.1				*Delftia* sp. (98%)	
	XP_005974267.1				*Acinetobacter* sp.	
	GL180697.1: 4765-5075^a^	*Xenopus tropicalis*	A	D	Comamonadaceae bacterium	ND
	SJM34589.1	*Homo sapiens* (Human)	A	D	*Mesorhizobium delmotii*	1
	XP_004001159.1	*Felis catus* (domestic cat)	A	D	*Acidovorax* sp. (97%)	1
	XP_019578089.1	*Rhinolophus sinicus* (Chinese rufous horseshoe bat)	A	D	*Brassica* sp. (94%)	1
	LVHJ01047219:4–240^a^	*Hippocampus comes* (Tiger tail seahorse)	U	A	Bacteroidetes bacterium (93.7%)	ND

*Abbreviations*: *ND* Not defined, *A* Available, *D* Discontinued, *U* Unavailable (Supplementary file 1)

^a^: Genomic location in the Ensembl genome browser 95

^b^: The sequencing shows only the first highly conserved sequence (CXDXR)

Our further analysis revealed that the genomic organization of the coding genes for the “vertebrate” β- and γ-CA proteins was consistent with the single exonic pattern of coding genes in prokaryotes. In addition, the BLAST homology search analysis decrypted the high percentage of identities (73–100%) between the predicted β- and γ-CA protein sequences of vertebrates and some other organisms, which mostly involved prokaryotic species (Table 1).

### Molecular analysis of *β-* and *γ-CA* genes from vertebrates

**Fig. 4:**
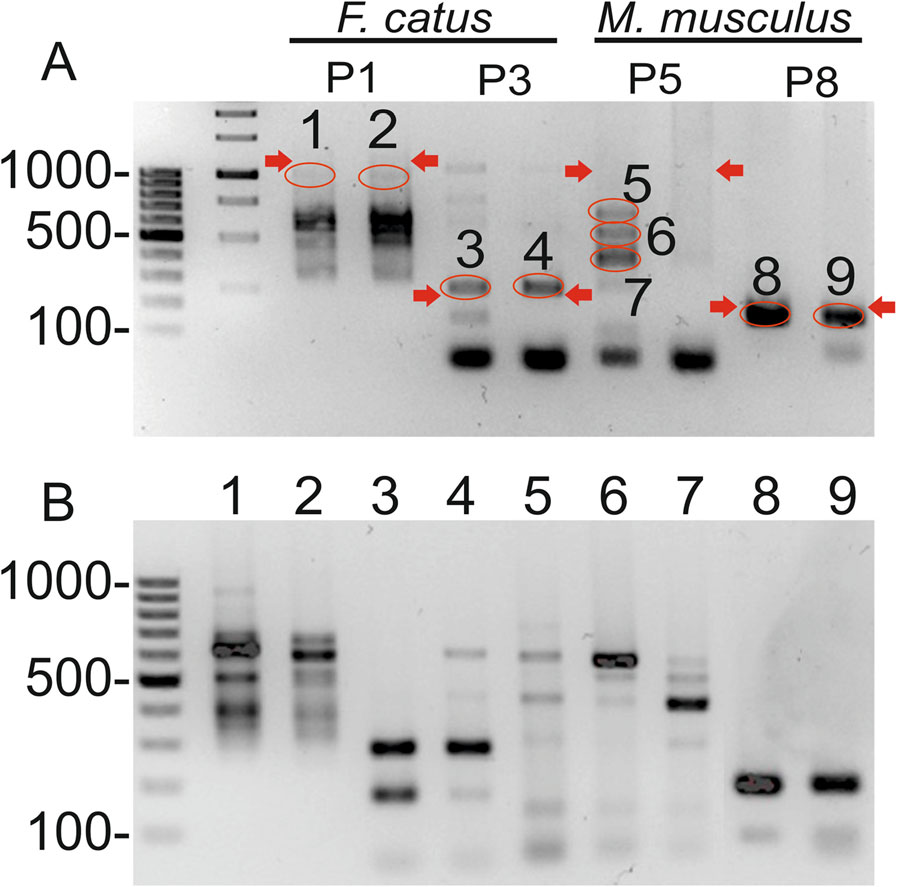
PCR analysis of the *γ-CA* gene from *F. catus* and *β-CA* gene from *M. musculus*. Samples from two animals of both species were included in the analysis, and primer pairs P1, P3, P5, and P8 were selected based on preliminary experiments. **a** shows the results from the first round of PCR. The bands nearest to the estimated correct size (red arrows) are marked with red circles (1–9). These bands were isolated, and the purified DNAs were used as templates for the second round of PCR. The results are shown in **b**. The amplified products from samples 3, 4, 8, and 9 were subsequently subjected to DNA sequencing

To investigate whether *β-CA* or *γ-CA* genes are truly present in vertebrate genomes, we performed PCR using DNA samples extracted from ear punching specimens of *M. musculus* and whole blood of *F. catus*. The first round PCRs with low stringent conditions showed some positive signal for the primer pairs P1 and P3 of *F. catus* and P5 and P8 of *M. musculus* (Fig. 4a). Estimation of the PCR product size was conducted based on the product length from Table 2. Because the signal remained weak in most cases, we performed the second round PCR using the PCR amplicons from the first round PCR as templates. The results of the second round of PCR are shown in Fig. 4b. The sequencing results revealed that none of the sequenced PCR products represented the predicted *β-CA* gene from *M. musculus* or the *γ-CA* gene from *F. catus*.

**Table 2:** Designed primers for the *β-* and *γ-CA* genes

CA family	Vertebrate species	Primer pairs		Product length (bp)
γ-CA	*Felis catus* (cat)	P1	Forward: 5′- AGATAACTACTTCACATCTGACA −3’	1089
			Reverse: 3′- ATACAGGGCTGGGTGCCT −5’	
		P2	Forward: 5′- GGTGATTGGCGACTACGTGA − 3’	625
			Reverse: 3′- CTCAGTCGGTTAGGTGGCTG − 5’	
		P3	Forward: 5′- GCGCGTGAAGAACAACTACC − 3’	217
			Reverse: 3′- GTGTTCAGTTGCGTCATCGG − 5’	
		P4	Forward: 5′- AAGCGGCAACCTCTACATCG −3’	341
			Reverse: 3′- CGTGAGGTAGGCAGTAGACG −5’	
β-CA	*Mus musculus* (Mouse)	P5	Forward: 5′- TGATAATGCCGATGGTCGTG −3’	1023
			Reverse: 3′- AGTAGCCATGGCCTTGCGAT −5’	
		P6	Forward: 5′- TGGATTTTCCGGCACCGTTA −3’	441
			Reverse: 3′- CGGGTCTTCCTTGCTGATGT −5’	
		P7	Forward: 5′- ACATCAGCAAGGAAGACCCG −3’	391
			Reverse: 3′- CACAATACGTCAAGGCGCTG −5’	
		P8	Forward: 5′- GCTGCACATCCGTGATCTCT −3’	191
			Reverse: 3′- GGATCCCATACACCCAACCG −5’	

## Discussion

*CA* genes are widely distributed in species of all life kingdoms. Despite this general concept, *β-* and *γ-CA* genes have never been reported in vertebrate genomes to the best of our knowledge based on previous literature. Our survey on the *β-* and *γ-CA* gene sequences of vertebrates presented in public databases in 2017–2020 revealed, however, that some sequences were or are still available, such as *β-CA* genes from *L. vexillifer* and *M. musculus*, as well as *γ-CA* genes from *L. vexillifer*. Some data were removed in 2019–2020, such as *β-CA* genes from *P. hodgsonii* and *H. sapiens*, as well as *γ-CA* genes from *P. hodgsonii, X. tropicalis, H. sapiens, F. catus,* and *R. sinicus*. Some new sequences appeared and were annotated on databases in 2019–2020, including *β-CA* genes from *H. comes, H. hucho,* and *O. tshawytscha*, as well as the *γ-CA* gene from *H. comes*. At first glance, the reports of “vertebrate” *β-* and *γ-CA* genes in databases raised our interest as a potentially novel discovery, but enthusiasm gradually dissipated as most data were discontinued in 2019–2020. The BLAST homology search analysis of the predicted “vertebrate” β- and γ-CA protein sequences filtered with the “prokaryota” keyword defined that the discontinued *β-* and *γ-CA* genes belonged to prokaryotes. The most striking false-positive sequences in databases were originally annotated as human β- and γ-CAs, which we defined by the BLAST homology search as *Mesorhizobium delmotii* enzymes instead of human origin (Table 1). Our results suggest that the predicted “human” β- and γ-CAs were derived from bacterial contamination of human DNA samples that caused false interpretation during sequencing. As a sign of improved accuracy, these false-positive data were removed from databases in 2019–2020.

Another piece of evidence for the bacterial contamination of DNA samples is the contamination of *H. comes* sample with *Muricauda* sp. and *Bacteroides* sp., both of which are abundantly present in seawater sediments [20, 21]. In addition, DNA samples of salmon fishes (*H. hucho* and *O. tshawytscha*) can be contaminated with gut microbiota or egg-associated bacterial species, such as *Flavobacterium* sp., *Pseudomonas* sp., and *Hydrogenophaga* sp. [22, 23]. *Comamonadaceae* bacterium from gut microbiota may represent the main source of bacterial contamination for the DNA samples of *X. tropicalis* [24]. Notably, due to the living habitat of *R. sinicus* in meadows, scrubs, and grasslands and feeding in these important ecosystems, the contamination of the bat DNA sample was mainly derived from plant species, such as *Brassica* sp. (cruciferous vegetables), instead of contamination from gut microbiota.

The exon count of the predicted “vertebrate” *β-* and *γ-CA* genes suggested the presence of only a single exon in each case. This finding also supported the idea that prokaryotes from gut microbiota and environmental microbiome are the major source of contaminants that led to unexpected sequencing results from vertebrate DNA samples [25]. This idea was further supported by our PCR analysis of both mouse and cat genomic DNA samples combined with DNA sequencing, which consistently failed to identify any *β-* or *γ-CA* sequences in mice and cats.

It is clear that a significant amount of incorrect sequence data on both *β-CA* and *γ-CA* genes remain in public databases. Some existing examples are *β-CA* genes of *L. vexillifer, M. musculus*, *H. comes, H. hucho*, and *O. tshawytscha* and *γ-CA* genes of *L. vexillifer* and *H. comes*. The present findings highlight the importance of database curation efforts to achieve a higher degree of accuracy within a shorter revision time.

## Conclusions

Online databases are important sources of information for mining genomic and proteomic data of living organisms. Unfortunately, these databases also include misannotated data to some extent due to microbial or other contamination. We used *β-* and *γ-CA* gene sequences as bioinformatic tools to demonstrate such contamination in various species. Our findings emphasize the importance of fast and reliable curation for achieving better-quality and more accurate genomic and proteomic data.

## Methods

### Identification of β- and γ-CAs

In the first step, the β- and γ-CA protein sequences from *Escherichia coli* (NCBI IDs: WP_000658644.1 and WP_131199889.1, respectively) were used as the query in the Basic local alignment search tool (BLAST) for sequence similarity search analysis through the BLASTP program (https://blast.ncbi.nlm.nih.gov/Blast.cgi?PAGE=Proteins) of NCBI database [26] and TBLASTN program of Ensembl genome browser 95 (https://asia.ensembl.org/Multi/Tools/Blast?db=core) [27]. We filtered the results using “vertebrata” as the organism name, in which the BLASTP program only searched for β- and γ-CA protein sequences within vertebrates. Additionally, we applied the scientific name of defined vertebrates as the filter in the TBLASTN program of Ensembl genome browser 95. The obtained β- and γ-CA protein sequences were aligned using the Clustal Omega algorithm (https://www.ebi.ac.uk/Tools/msa/clustalo/) [28].

In the second step, we performed a BLAST homology search analysis on the obtained β- and γ-CA protein sequences from vertebrates, in which the results were filtered against “prokaryota” as the organism name. Afterward, exon counts were performed to detect *β-* and *γ-CA* gene sequences from vertebrates through the gene analysis program of the NCBI database.

### Molecular analysis of *β-* and *γ-CA* genes from vertebrates

We designed eight primer pairs using Primer-BLAST for molecular detection of the *β-CA* gene from *Mus musculus* (Mouse) and the *γ-CA* gene from *Felis catus* (cat) (four primer pairs for each *CA* gene) identified through bioinformatic methods (Table 2) [29].

The ear blood samples of one *M. musculus* and 1 ml EDTA-blood samples of one privately-owned *F. catus* were collected under the permission of the animal ethical committee of the County Administrative Board of Southern Finland (ESAVI/8321/04.10.07/2017 for the mouse and ESAVI/7482/04.10.07/2015 for the cat) for molecular detection of the predicted *β-CA* gene of *M. musculus* and *γ-CA* gene of *F. catus.* In the Tampere University’s animal facility, mice are routinely earmarked and the same samples were used for genotyping purposes in another project. Written consents were collected from the participating cat owners and samples were collected as a part of the ongoing feline genetic research at Dr. Lohi’s laboratory. Cats visited a veterinary clinic for a routine sample collection. Genomic DNA was extracted from white blood cells using a semiautomated Chemagen extraction robot (PerkinElmer Chemagen Technologie GmbH, Baeswieler, Germany) according to the manufacturer’s instructions. The DNA concentrations were measured using a Qubit fluorometer (Thermo Fisher Scientific, Waltham, Massachusetts, USA) and a Nanodrop ND-1000 UV/Vis Spectrophotometer (Nanodrop Technologies, Wilmington, Delaware, USA), and samples were stored at − 20 °C. Polymerase chain reaction (PCR) was performed according to the protocol used by Zolfaghari Emameh R et al. [30]. PCR amplification was run on a thermocycler (Bioer XP Cycler, Hangzhou Bioer Technology Co. Ltd., Hangzhou, China) according to the following details: 95 °C (3 min), [95 °C (15 s), 60 °C (15 s), 72 °C (15 s)] × 40 cycles, 72 °C (2 min). The amplified products were run on a 1.6% agarose gel and purified using a NucleoSpin Gel and PCR Clean-up kit (Macherey-Nagel). The second round of PCR was run as previously described, and the selected PCR amplicons (Fig. 4; samples 3, 4, 8, and 9) were treated with Exo I and Fast AP enzymes and sequenced using ABI PRISM BigDye® Terminator v3.1 Cycle Sequencing kit and 3500xL Genetic Analyzer (Applied Biosystems, Inc., Foster City, CA, U.S.A.).

## Supplementary information

**Additional file 1.** Supplementary file 1

## Data Availability

No novel DNA, RNA, and protein sequence data related to β- and γ-CAs were produced in this study to be annotated in the databases. The analyzed datasets used in the current study were collected from NCBI database including XP_007454654.1, XP_007466906.1, XP_005974256.1, XP_005956696.1, XP_005973271.1, XP_005979975.1, XP_005954808.1, SJM31717.1, XP_024266887.1, XP_007452618.1, XP_007465530.1, XP_005974442.1, XP_005977566.1, XP_005974267.1, SJM34589.1, XP_004001159.1, and XP_019578089.1 as well as Ensembl genome browser including LVXS01065484.1: 870–1,430, LVHJ01039623:18–230, QNTS01034426:189–644, GL180697.1: 4,765-5,075, and LVHJ01047219:4–240.

## Abbreviations

BLAST: Basic Local Alignment Search Tool
CA: Carbonic anhydrase
CARP: CA-related protein
MSA: Multiple sequence alignment
NCBI: National Center for Biotechnology Information
PCR: Polymerase chain reaction

## References

[1] Del Prete S, Vullo D, Fisher GM, Andrews KT, Poulsen SA, Capasso C (2014). Discovery of a new family of carbonic anhydrases in the malaria pathogen Plasmodium falciparum--the eta-carbonic anhydrases. Bioorg Med Chem Lett.

[2] Kikutani S, Nakajima K, Nagasato C, Tsuji Y, Miyatake A, Matsuda Y (2016). Thylakoid luminal theta-carbonic anhydrase critical for growth and photosynthesis in the marine diatom Phaeodactylum tricornutum. Proc Natl Acad Sci U S A.

[3] Jensen EL, Clement R, Kosta A, Maberly SC, Gontero B (2019). A new widespread subclass of carbonic anhydrase in marine phytoplankton. ISME J..

[4] Del Prete S, Nocentini A, Supuran CT, Capasso C (2020). Bacterial iota-carbonic anhydrase: a new active class of carbonic anhydrase identified in the genome of the gram-negative bacterium Burkholderia territorii. J Enzyme Inhib Med Chem.

[5] Supuran CT (2008). Carbonic anhydrases: novel therapeutic applications for inhibitors and activators. Nat Rev Drug Discov.

[6] Reibring CG, El Shahawy M, Hallberg K, Kannius-Janson M, Nilsson J, Parkkila S (2014). Expression patterns and subcellular localization of carbonic anhydrases are developmentally regulated during tooth formation. PLoS One.

[7] Hilvo M, Tolvanen M, Clark A, Shen B, Shah GN, Waheed A (2005). Characterization of CA XV, a new GPI-anchored form of carbonic anhydrase. Biochem J.

[8] Aspatwar A, Tolvanen ME, Ojanen MJ, Barker HR, Saralahti AK, Bauerlein CA (2015). Inactivation of ca10a and ca10b genes leads to abnormal embryonic development and alters movement pattern in Zebrafish. PLoS One.

[9] Sterky FH, Trotter JH, Lee SJ, Recktenwald CV, Du X, Zhou B (2017). Carbonic anhydrase-related protein CA10 is an evolutionarily conserved pan-neurexin ligand. Proc Natl Acad Sci U S A.

[10] Karjalainen SL, Haapasalo HK, Aspatwar A, Barker H, Parkkila S, Haapasalo JA (2018). Carbonic anhydrase related protein expression in astrocytomas and oligodendroglial tumors. BMC Cancer.

[11] Ogilvie JM, Ohlemiller KK, Shah GN, Ulmasov B, Becker TA, Waheed A (2007). Carbonic anhydrase XIV deficiency produces a functional defect in the retinal light response. Proc Natl Acad Sci U S A.

[12] Frost SC (2014). Physiological functions of the alpha class of carbonic anhydrases. Subcell Biochem.

[13] Zolfaghari Emameh R, Barker HR, Tolvanen ME, Parkkila S, Hytonen VP (2016). Horizontal transfer of beta-carbonic anhydrase genes from prokaryotes to protozoans, insects, and nematodes. Parasit Vectors.

[14] Zolfaghari Emameh R, Barker HR, Hytonen VP, Parkkila S (2018). Involvement of beta-Carbonic Anhydrase Genes in Bacterial Genomic Islands and Their Horizontal Transfer to Protists. Appl Environ Microbiol.

[15] Ferry JG (2010). The gamma class of carbonic anhydrases. Biochim Biophys Acta.

[16] Zolfaghari Emameh R, Barker HR, Syrjanen L, Urbanski L, Supuran CT, Parkkila S (2016). Identification and inhibition of carbonic anhydrases from nematodes. J Enzyme Inhib Med Chem.

[17] Syrjanen L, Tolvanen M, Hilvo M, Olatubosun A, Innocenti A, Scozzafava A (2010). Characterization of the first beta-class carbonic anhydrase from an arthropod (Drosophila melanogaster) and phylogenetic analysis of beta-class carbonic anhydrases in invertebrates. BMC Biochem.

[18] Lu J, Salzberg SL (2018). Removing contaminants from databases of draft genomes. PLoS Comput Biol.

[19] Dittami SM, Corre E (2017). Detection of bacterial contaminants and hybrid sequences in the genome of the kelp Saccharina japonica using Taxoblast. PeerJ..

[20] Huntemann M, Teshima H, Lapidus A, Nolan M, Lucas S, Hammon N (2012). Complete genome sequence of the facultatively anaerobic, appendaged bacterium Muricauda ruestringensis type strain (B1(T)). Stand Genomic Sci.

[21] Fernandez-Gomez B, Richter M, Schuler M, Pinhassi J, Acinas SG, Gonzalez JM (2013). Ecology of marine Bacteroidetes: a comparative genomics approach. ISME J.

[22] Dehler CE, Secombes CJ, Martin SA (2017). Environmental and physiological factors shape the gut microbiota of Atlantic salmon parr (Salmo salar L.). Aquaculture..

[23] Wilkins LG, Rogivue A, Schutz F, Fumagalli L, Wedekind C (2015). Increased diversity of egg-associated bacteria on brown trout (Salmo trutta) at elevated temperatures. Sci Rep.

[24] Colombo BM, Scalvenzi T, Benlamara S, Pollet N (2015). Microbiota and mucosal immunity in amphibians. Front Immunol.

[25] Goig GA, Blanco S, Garcia-Basteiro AL, Comas I (2020). Contaminant DNA in bacterial sequencing experiments is a major source of false genetic variability. BMC Biol.

[26] Johnson M, Zaretskaya I, Raytselis Y, Merezhuk Y, McGinnis S, Madden TL (2008). NCBI BLAST: a better web interface. Nucleic Acids Res.

[27] Fernandez-Suarez XM, Schuster MK (2010). Using the ensembl genome server to browse genomic sequence data. Curr Protoc Bioinformatics.

[28] Sievers F, Higgins DG (2018). Clustal omega for making accurate alignments of many protein sequences. Protein Sci.

[29] Ye J, Coulouris G, Zaretskaya I, Cutcutache I, Rozen S, Madden TL (2012). Primer-BLAST: a tool to design target-specific primers for polymerase chain reaction. BMC Bioinformatics.

[30] Zolfaghari Emameh R, Kuuslahti M, Nareaho A, Sukura A, Parkkila S (2016). Innovative molecular diagnosis of Trichinella species based on beta-carbonic anhydrase genomic sequence. Microb Biotechnol.

