# Supplementary material for: Assessment of databases to determine the validity of β- and γ-carbonic anhydrase sequences from vertebrates
Source: BMC Genomics. 2020 May 11;21:352. doi: 10.1186/s12864-020-6762-2 (PMC7216627; doi:10.1186/s12864-020-6762-2)

**Supplementary file 1**

1. **The NCBI IDs that discontinued by the curation system of database:**

>XP_005974256.1 PREDICTED: carbonic anhydrase 2, chloroplastic-like [Pantholops hodgsonii]

MKDFPETLLNGYKNFMSGRYVDERERYRVLADTGQKPQTLFIACCDSRSAPETIFDCGPGELFVVRNVAN

MVPPFEPDGQYHATSAAIEYAVQVLKVKDIVVMGHGRCGGIQAALDPNLEPLSPGDFIGKWMNMVKSAAE

QIQSNDVMTASERQTALERVSIRTSIANLRGFPFVKAQETAGKVKLHGAWFDISTGELWVMDGKTGDFRR

PDL

>XP_005956696.1 PREDICTED: carbonic anhydrase, chloroplastic-like [Pantholops hodgsonii]

MTDFADMLEGYRRFRDTGWSQQRERWDELNEGQSPKVMVIACSDSRVDPAQIFDTSPGEIFVVRNVAALV

PPFETNPGWHGVSAALEFAVQVLKVGEIVVMGHGKCGGCKAALSHDLKDAPPGEGGFIHNWIQLLDDARD

VVVGQYGDQRDRDVERAMEQEGVKVSLTNLRSFPCVRAKEKSGELKLVGSFFAISDGQLHILDEQTGVFA

AA

>XP_005973271.1 PREDICTED: carbonic anhydrase 2, chloroplastic-like [Pantholops hodgsonii]

MPHLPEHLLAGYRSFINGRYVAESGHYKSLAREGQAPETMIVACCDSRAAPEVIFNAGPGELFVLRNVAN

LVPPYAPDGEYHSTSAALEFAVQSLKVKNIVVMGHGRCGGIRSALDTTSAPLSPGDFIGKWMSLIAPAAE

AVSASTMMTTTERQTALERISIRYSIANLRTFPCVSILEGKGRIALHGAWFDISTGELWVMNKETGDFEK

PDIG

>XP_005979975.1 PREDICTED: carbonic anhydrase 2-like [Pantholops hodgsonii]

MLTAQEALERLKQGNERFAHGETNLTKFLTHQQRSEMMDSQEPFAIILGCSDSRVPAEMVFDQGFGDLFV

IRVAGNIVAPSQVGSVEFAADSFGCPLVVVLGHSHCGAIHSTIEALKNPNTPPSANLMSIVNRVRPSVEI

LMQTELKDDLEKLSMHAVKSNVFASVNQLRHGSAVLENLIAKGKLKVVGAEYSLETGEVSFYDF

>XP_005954808.1 PREDICTED: carbonic anhydrase, chloroplastic-like [Pantholops hodgsonii]

MAKSGVGQAGLHRIVGGASGLGLSIALRKPVRHHGASSSQGDRPMCSDVDGMHDQPHGTQEEDGAAPSRR

AFLTAMTMAGGVAMAALPSVSAMAAPPADGPDASMTPDAALAEIMAGNARFVAGKPTAHLQDLSIIKARA

AEGQWPVVGVLSCADSRVPVEMVFDEYIGRLFVTRIAGNITTPEIVASLEYGVAVLGLKALVVMGHSNCG

AVKAAIDNPEVPGQISALFPAILPALYLARSKDAAIVTRTNALVQAATLVNASPVIEEKVKAGTLKVVAA

VYDVATGKVDMLPVPADMLMRG

>XP_005974442.1 PREDICTED: gamma carbonic anhydrase 3, mitochondrial-like [Pantholops hodgsonii]

MTVYSLGAVAPTLPPQDEYWIAPTASVMGNVVLKRNASIWWGAVARGDNDPITIGENSNVQDGSVLHTDL

GFPLTIGANVTIGHMVMLHGCAIGDGSLIGIGAVVLNGARIGKNCLIGAGALITEGKEIPDNSMVMGAPG

KVVREVSEHHAQILQGSALHYVENWKRYARELRAVEG

>XP_005977566.1 PREDICTED: gamma carbonic anhydrase 2, mitochondrial-like [Pantholops hodgsonii]

MAIYELDGVAPEVAGGGVADSAEVMGNVQLAEDASIWFGAVLRGDCESITIGEGSNIQDASVLHADLGQP

LVVGRHVTVGHQVMLHGCTIGDESLIGIGAVVLNGAKIGRNCLVGAGALITEGKEFPDGSMIIGSPAKAV

RQLTPEQIEGLRRSAQHYVDNARRFKTGLRKLG

>XP_005974267.1 PREDICTED: gamma carbonic anhydrase 2, mitochondrial-like [Pantholops hodgsonii]

MYKFKGFVPKALHAPFDGWIADNATVIGQVELGQQVSVWFGAVIRADNAKIHIGNFSNVQENAVLHTDTG

IELTVGDYVTIGHQAMLHGCTIGDNSLIGINAVVLNHAVIGKNCIIGANSLIPEGKVIPDNSLVVGSPGK

VVRTLDENAENLLKMSALHYAAHYKNFIDLEEFKFD

>XP_004001159.1 PREDICTED: uncharacterized protein LOC101091771, partial [Felis catus]

FLSNGQKTHSSAQVIGDVQLGDDSSVWCNAVLRGDVNRITVGRCSNVQDLTMGHVSHRNASKPEGSPLVI

GDYVTVGHSVILHGCRIGNECLIGMGSIVMDDAVIEDRVMLGAGSLVPPGKVLESGNLYIGRPAVRQRAL

TEAEIAYLKYSAEHYVRVKNNYLAGSPAAPLPEAAASKGQGTPG

>XP_019578089.1 PREDICTED: gamma carbonic anhydrase 1, mitochondrial [Rhinolophus sinicus]

MGTLGRAFYSVGFWIRETGQALDRLGCRLQGKNCFREQLSRHRTLMNVFDKAPIVDKEAFVAPSASVIGN

VQIGRGSSIWYGCVLRGDVNTVSVGSGTNIQDNSLVHVAKSNLSGKVPATTIGDNVTIGHSAVLHGCTVE

DEAFIGMGATLLDGVVVEKHGMVAAGALVRQNTRIPSREIWGGNPARFLRKLRDEEIDFFPKSAANYSNL

AKVHAAENAKPLNAVEFEKVLRKKYARKDEEYDSMLGIVRETPPELKLPNNIEPAKEAKRPSNVN

1. **The Ensembl ID that discontinued by the curation system of database:**

- The available ensemble data for “GL180697.1” (γ-CA of *X. tropicalis*) is the following sequence and figure:

>GL180697.1 PREDICTED: [Xenopus tropicalis]

YAFEGLRPVVHPTAYVHPEATLIGDVIVGPGCYLAPQASLRGDYGRLIVEAGANVQDGCIMHGYDKVDTV

VGPGASIGHGAILHGCRIGAGSVVGM


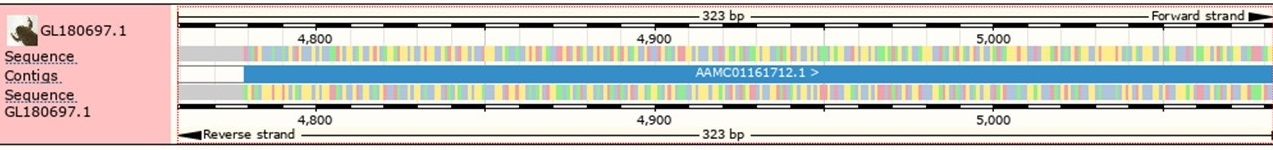

Supplement: Supplementary file 1 — Additional file 1. Supplementary file 1 [file 12864_2020_6762_MOESM1_ESM.doc]
